# Platypnea-Orthodeoxia Syndrome after Transcatheter Aortic Valve Implantation

**DOI:** 10.1155/2016/6954121

**Published:** 2016-08-17

**Authors:** Andrew K. Roy, Jerome Garot, Antoinette Neylon, Marco Spaziano, Fadi J. Sawaya, Thierry Lefèvre

**Affiliations:** Institut Cardiovasculaire Paris-Sud, Hôpital Privé Jacques Cartier, 91300 Massy, France

## Abstract

Progressive dyspnea and hypoxaemia in the subacute phase after transcatheter aortic valve implantation (TAVI) are uncommon and warrant immediate assessment of valve and prosthesis leaflet function to exclude thrombosis, as well as investigation for other causes related to the procedure, such as left ventricular dysfunction, pulmonary embolism, and respiratory sepsis. In this case, we report the observation of a patient presenting two weeks after TAVI with arterial hypoxaemia in an upright position, relieved by lying flat, and coupled with an intracardiac shunt detected on echocardiography in the absence of pulmonary hypertension, raising the suspicion of Platypnea-Orthodeoxia Syndrome (POS). Invasive intracardiac haemodynamic assessment showed a significant right-to-left shunt (Qp/Qs = 0.74), which confirmed the diagnosis, with subsequent closure of the intracardiac defect resulting in immediate relief of symptoms and hypoxaemia. To our knowledge, this is the first reported case of an interatrial defect and shunt causing Platypnea-Orthodeoxia Syndrome after transcatheter aortic valve implantation, resolved by percutaneous device closure.

## 1. Introduction

Dyspnea is the commonest presenting symptom for severe calcific aortic stenosis, occurring in approximately 60–70% of patients who are considered at high or prohibitive risk for surgery but eligible for TAVI [1]. For these patients, TAVI significantly improves mortality when compared with medical therapy for aortic stenosis and leads to sustained improvement of cardiac symptoms and in the majority of patients [1].

To determine suitability for TAVI under conscious sedation, patients will typically undergo a number of investigations including transthoracic echocardiography, coronary angiography, thoracic multidetector computed tomography, and assessment of the peripheral vasculature for iliofemoral patency. During this evaluation, any other reversible cardiac causes of dyspnea, such as severe coexisting proximal coronary artery disease or congestive heart failure, will be detected and treated with the aim of minimizing periprocedural events and ensuring an optimal and durable response to TAVI. Thus, the development of acute or subacute dyspnea in the initial period after TAVI is a serious concern, prompting the clinician to immediately consider mechanical aortic prosthesis dysfunction, as a result of thrombosis or infection, along with other more common causes of dyspnea such as congestive heart failure, pulmonary embolism, and respiratory sepsis. In this case report of new onset dyspnea one week after TAVI, the observation of postural hypoxaemia, normal aortic prosthesis function, and clear evidence of a new intracardiac shunt on echocardiography prompted us to consider the rare clinical Platypnea-Orthodeoxia Syndrome as a cause for this patient's symptoms.

## 2. Case Presentation

**Figure 1:**
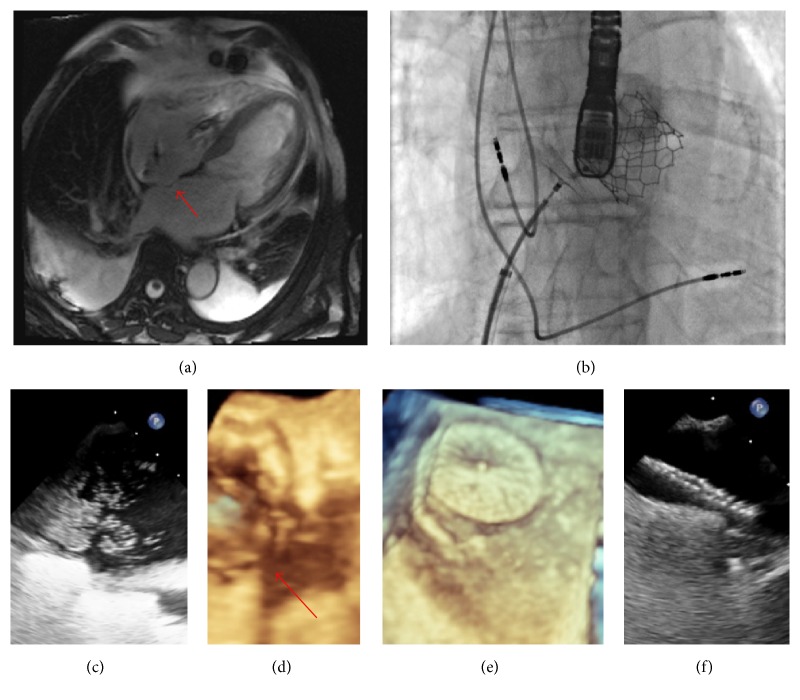
Platypnea-Orthodeoxia Syndrome after transcatheter aortic valve implantation. (a) Cardiac Magnetic Resonance 4-Chamber Image from 4 years earlier, showing thinned and bulging interatrial septum (red arrow) with no evidence of aneurysm or transseptal contrast flow, right ventricular thickness = 7 mm, and mild tricuspid incompetence with no evidence of carcinoid infiltration. (b) Fluoroscopy imaging showing 35 mm septal occlusion device in situ before deployment, with Edwards S3*™* 29 mm transcatheter aortic valve device and transoesophageal probe visible. (c) Intraoperative transoesophageal contrast study showing a large shunt from right-to-left atrium, possibly with the presence of more than one fenestrated defect. (d) Three-dimensional transoesophageal image of defect (red arrow) and transcatheter aortic valve in situ. (e) Three-dimensional transoesophageal showing PFO device in situ. (f) Postprocedure negative bubble study with device in situ.

A 65-year-old male with severe symptomatic aortic stenosis, pacemaker, mild left ventricular dysfunction, and metastatic carcinoid syndrome underwent transcatheter aortic valve implantation (TAVI) under conscious sedation with an Edwards S3*™* 29 mm device. His postoperative course was unremarkable and he was discharged well 7 days later. One week after discharge, he represented to our center with progressive dyspnea and resting hypoxaemia, with standing oxygen saturation of 82%, not responding adequately to supplemental oxygen, but returning to 97% by lying flat. Pulmonary scintigraphy was negative for pulmonary embolism and there was no evidence of respiratory or systemic sepsis. N-Terminal pro-Brain Natriuretic Peptide was elevated at 803 pg/mL (pre-TAVI = 3400 pg/mL, discharge post-TAVI = 400 pg/mL). Immediate transthoracic echocardiography was performed, which did not reveal TAVI prosthesis dysfunction or aortic regurgitation but did show moderate right atrial dilatation. The mean transaortic gradient was 6 mmHg, unchanged from that measured immediately after TAVI. Transoesophageal contrast echocardiography revealed a bulging interatrial septum, along with a significant right-to-left interatrial shunt (Figure 1) that was not present when compared to previous magnetic resonance imaging (Figure 1). Right ventricular function was normal, with normalization of his left ventricular function. Cardiac catheterization revealed the following haemodynamic measurements: right atrial pressure, 11 mmHg; mean pulmonary artery pressure, 18 mmHg; pulmonary artery capillary wedge pressure, 9 mmHg; and cardiac output, 4.6 L/min. Oxygen saturations were measured as follows: inferior vena cava, 65.0%; superior vena cava, 74.1%; low right atrium, = 72.3%; high right atrium, 68.7%; pulmonary artery, 70.4%; left atrium, 87.6%; left superior pulmonary vein, 97.0%; and aorta, 91.5%, giving a Qp/Qs shunt ratio of 0.74, consistent with a right-to-left shunt. Based upon the demonstration of an anatomical interatrial defect, functional shunting in the setting of recent TAVI, and clinical symptoms with impaired oxygenation, the diagnosis of Platypnea-Orthodeoxia Syndrome was made and we elected to close the PFO. The patient's symptoms resolved with septal occlusion using a 35 mm Patent Foramen Ovale (PFO) occlusion device (Figures 1(c)–1(f)), and no hypoxia was observed at discharge the following day. At one-year follow-up the patient was well with no recurrence of symptoms. To our knowledge, this is the first case of symptomatic atrial shunting across PFO described after TAVI.

## 3. Discussion

Platypnea-Orthodeoxia Syndrome (POS) is an uncommon clinical cause of dyspnea and hypoxaemia associated with interatrial communications and functional alterations required to preferentially shunt venous return towards the septal defect [2]. It is associated with a number of cardiac, pulmonary, or abdominal conditions and, given the potentially reversible nature, should always be considered when hypoxaemia with postural features remains unresponsive to standard oxygen therapy [3]. For patients with aortic disease, the aorta may compress the right atrium, and POS has been described in the setting of dilated or horizontal aortas due to aortic valve disease, aneurysms, kyphoscoliosis, previous surgical repair, and congenital heart defects [3]. POS has also been described in a patient who had undergone recent surgical aortic valve replacement, attributed to the presence of significant aortic dilatation [2–4].

The diagnosis is confirmed with cardiac imaging to visualize the septal defect, ideally by transoesophageal echocardiography with bubble/contrast study, although cardiac magnetic resonance imaging or right heart catheterization and shunt calculation are also suitable, particularly where the diagnosis is unclear. The definitive treatment is septal occlusion using a percutaneous occlusion device or surgical approach. For the patient in this case report, it is postulated that a state of chronic shunt equalization (as indicated by septal bulging with no shunt on previous cardiac MRI) was stable until TAVI was performed, with left ventricular compliance and diastolic pressures rapidly improving, causing a situation where venous return from the IVC, with distortion of the right atrium, was now preferentially shunted across the PFO when the patient was upright.

As TAVI is rapidly becoming accepted as a standard treatment for aortic stenosis, often for patients who are older, with more aortic dilatation, and other comorbid conditions that may predispose to Platypnea-Orthodeoxia Syndrome, clinicians should consider this potentially reversible condition when unexplained hypoxaemia and dyspnea present in the setting of aortic valve disease.
